# Non contiguous-finished genome sequence and description of *Bacillus timonensis* sp. nov.

**DOI:** 10.4056/sigs.2776064

**Published:** 2012-07-20

**Authors:** Sahare Kokcha, Ajay Kumar Mishra, Jean-Christophe Lagier, Matthieu Million, Quentin Leroy, Didier Raoult, Pierre-Edouard Fournier

**Affiliations:** 1 Unité de Recherche sur les Maladies Infectieuses et Tropicales Emergentes, UMR CNRS 6236 – IRD 198, Faculté de médecine, Aix-Marseille Université

**Keywords:** *Bacillus timonensis*, genome

## Abstract

*Bacillus timonensis* strain MM10403188^T^ sp. nov. is the type strain of a proposed new species within the genus *Bacillus*. This strain, whose genome is described here, was isolated from the fecal flora of a healthy patient. *B. timonensis* is an aerobic Gram-negative rod shaped bacterium. Here we describe the features of this organism, together with the complete genome sequence and annotation. The 4,632,049 bp long genome (1 chromosome but no plasmid) contains 4,610 protein-coding and 74 RNA genes, including 5 rRNA genes.

## Introduction

*Bacillus timonensis* strain MM10403188^T^ (= CSUR P162 = DSM 25372) is designated as the type strain of *B. timonensis*, a new Gram-negative aerobic, indole-positive bacillus that was isolated from the stool of a healthy Senegalese patient as part of a “culturomics” study aiming at cultivating individually all species within human feces.

To date, DNA-DNA hybridization and G+C content determination [1] remain the gold standard methods for the definition of bacterial species, despite the development of 16S rRNA PCR and sequencing which have deeply changed bacterial taxonomy [2]. Over recent years, high throughput genome sequencing provided a wealth of genetic information [3]. In an effort to include genomic data in bacterial taxonomy we recently used a polyphasic approach [4] that includes genomic data, MALDI-TOF spectrum and main phenotypic characteristics to describe new bacterial species [5,6] .

Here we present a summary classification and a set of features for *B. timonensis* sp. nov. strain MM10403188^T^ together with the description of the complete genomic sequencing and annotation. These characteristics support the circumscription of the species *B. timonensis*.

The genus *Bacillus* (Cohn 1872) was created in 1872 [6]. To date, this genus, mostly comprised of Gram-positive, motile, and spore-forming bacteria, is made of 256 species and 7 subspecies with validly published names [7]. Members of the genus *Bacillus* are ubiquitous bacteria isolated from various environments including soil, fresh and sea water, food, and occasionally from humans in whom they are either pathogens, such as *B. anthracis* and *B. cereus,* or opportunists in immunocompromised patients [7]. Apart from anthrax, caused by *B. anthracis* [8], and toxi-infections caused by *B. cereus*, *Bacillus* species may be involved in a variety of aspecific human infections, including cutaneous, ocular, central nervous system or bone infections, pneumonia, endocarditis and bacteremia [9].

## Classification and features 

**Figure 1:**
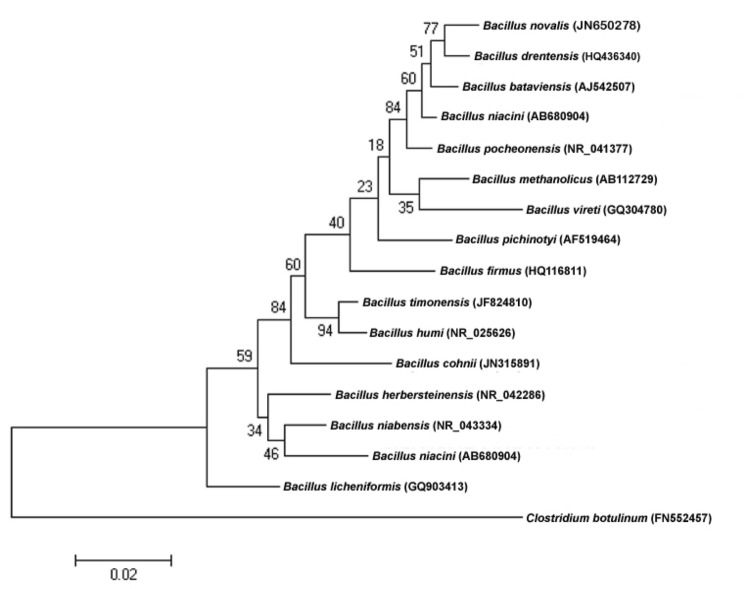
Phylogenetic tree highlighting the position of *Bacillus timonensis* strain MM10403188^T^ relative to other type strains within the *Bacillus* genus. GenBank accession numbers are indicated in parentheses. Sequences were aligned using CLUSTALW, and phylogenetic inferences obtained using the maximum-likelihood method within the MEGA software. Numbers at the nodes are percentages of bootstrap values obtained by repeating the analysis 500 times to generate a majority consensus tree. *Clostridium botulinum* was used as an outgroup. The scale bar represents a 2% nucleotide sequence divergence.

A stool sample was collected from a healthy 16-year-old male Senegalese volunteer patient living in Dielmo (a rural village in the Guinean-Sudanian zone in Senegal), who was included in a research protocol. The patient gave an informed and signed consent, and the agreement of the National Ethics Committee of Senegal and the local ethics committee of the IFR48 (Marseille, France) was obtained under agreements 09-022 and 11-017). The fecal specimen was preserved at -80°C after collection and sent to Marseille. Strain MM10403188 (Table 1) was isolated in June 2011 by cultivation on 5% sheep blood-enriched Brain Heart Infusion agar with (Becton Dickinson, Heidelberg, Germany). This strain exhibited a 98.2% nucleotide sequence similarity with *Bacillus humi*, the phylogenetically closest validated *Bacillus* species (Figure 1). This value was lower than the 98.7% 16S rRNA gene sequence threshold recommended by Stackebrandt and Ebers to delineate a new species without carrying out DNA-DNA hybridization [2].

**Table 1:** Classification and general features of *Bacillus timonensis* strain MM10403188^T^

**MIGS ID**	**Property**	**Term**	**Evidence code^a^**
	Current classification	Domain *Bacteria*	TAS [10]
		Phylum *Firmicutes*	TAS [11-13]
		Class *Bacilli*	TAS [14,15]
		Order *Bacillales*	TAS [16,17]
		Family *Bacillaceae*	TAS [16,18]
		Genus *Bacillus*	TAS [16,19,20]
		Species *Bacillus timonensis*	IDA
		Type strain MM10403188^T^	IDA
	Gram stain	negative	IDA
	Cell shape	rod	IDA
	Motility	motile	IDA
	Sporulation	sporulating	IDA
	Temperature range	mesophile	IDA
	Optimum temperature	37°C	IDA
MIGS-6.3	Salinity	growth in BHI medium + 5% NaCl	IDA
MIGS-22	Oxygen requirement	aerobic	IDA
	Carbon source	unknown	NAS
	Energy source	unknown	NAS
MIGS-6	Habitat	human gut	IDA
MIGS-15	Biotic relationship	Free living	IDA
MIGS-14	Pathogenicity Biosafety level Isolation	unknown 2 human feces	NAS
MIGS-4	Geographic location	Senegal	IDA
MIGS-5	Sample collection time	September 2010	IDA
MIGS-4.1	Latitude	13.7167	IDA
MIGS-4.1	Longitude	-16.4167	IDA
MIGS-4.3	Depth	Surface	IDA
MIGS-4.4	Altitude	51 m above sea level	IDA

Evidence codes - IDA: Inferred from Direct Assay; TAS: Traceable Author Statement (i.e., a direct report exists in the literature); NAS: Non-traceable Author Statement (i.e., not directly observed for the living, isolated sample, but based on a generally accepted property for the species, or anecdotal evidence). These evidence codes are from the Gene Ontology project [21]. If the evidence is IDA, then the property was directly observed for a live isolate by one of the authors or an expert mentioned in the acknowledgements.

**Figure 2:**
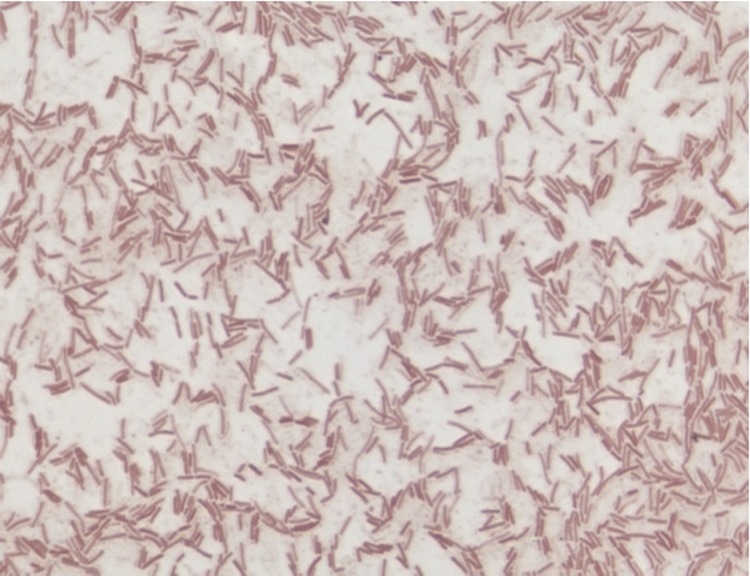
Gram staining of *B. timonensis* strain MM10403188^T^

**Figure 3:**
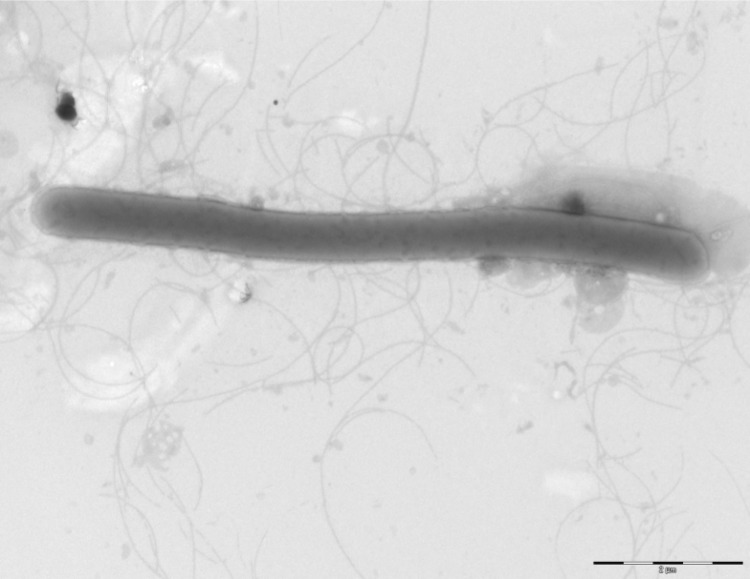
Transmission electron microscopy of *B. timonensis* strain MM10403188^T^, using a Morgani 268D (Philips) at an operating voltage of 60kV. The scale bar represents 900 nm.

Different growth temperatures (25, 30, 37, 45°C) were tested. Growth occurred at all tested temperatures, but optimal growth occurred between 30 and 37°C. Colonies were 3 mm in diameter on blood-enriched BHI agar. Growth of the strain was tested under anaerobic and microaerophilic conditions using GENbag anaer and GENbag microaer systems, respectively (BioMérieux), and in aerobic conditions, with or without 5% CO_2_. Growth was achieved in aerobic (with and without CO_2_) and microaerophilic conditions. No growth was observed in anaerobic conditions. Gram staining showed Gram negative bacilli (Figure 2). A motility test was positive. Cells grown on agar are sporulated and have a mean diameter of 0.66 µm (Figure 3).

Strain MM10403188^T^ exhibited oxidase activity but not catalase activity, and was positive for indole. Using API 50CH, a positive reaction was obtained for L-arabinose, D-lactose, D-melibiose, D-trehalose, D-saccharose, and D-turanose fermentation. A weak reaction was obtained for aesculin. Other tests were negative. Using API-ZYM, positive reactions were obtained for esterase, α-chimotrypsine, β-glucorinidase, and α- and β-glucosinidase. *B. timonensis* was susceptible to penicillin G, amoxicillin, vancomycin, gentamicin, erythromycin, doxycyclin, rifampicin, and ciprofloxacin but resistant to trimethoprim/sulfamethoxazole. 

By comparison with *B. humi*, *B. timonensis* differed in Gram staining, in culture atmosphere, as *B. humi* was able to grow anaerobically, in catalase activity, in spore forming capacity, in indole production, and in carbohydrate metabolism, notably for arbutin, salicin, L-arabinose, melibiose, turanose, and trehalose [22].

**Figure 4:**
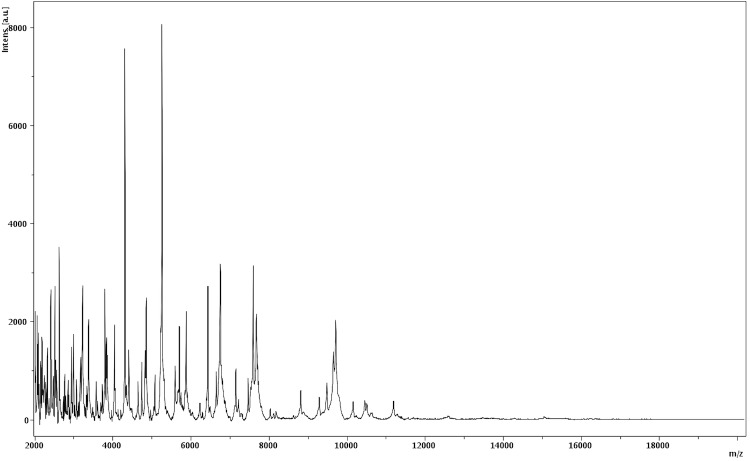
Reference mass spectrum from *B. timonensis* strain MM10403188^T^. Spectra from 12 individual colonies were compared and a reference spectrum was generated.

Matrix-assisted laser-desorption/ionization time-of-flight (MALDI-TOF) MS protein analysis was carried out as previously described [23]. Briefly, a pipette tip was used to pick one isolated bacterial colony from a culture agar plate, and to spread it as a thin film on a MTP 384 MALDI-TOF target plate (Bruker Daltonics, Leipzig, Germany). Four distinct deposits were done for strain MM10403188 from four isolated colonies. Each smear was overlaid with 2µL of matrix solution (saturated solution of alpha-cyano-4-hydroxycinnamic acid) in 50% acetonitrile, 2.5% tri-fluoracetic-acid, and allowed to dry for five minutes. Measurements were performed with a Microflex spectrometer (Bruker). Spectra were recorded in the positive linear mode for the mass range of 2,000 to 20,000 Da (parameter settings: ion source 1 (IS1), 20 kV; IS2, 18.5 kV; lens, 7 kV). A spectrum was obtained after 675 shots at a variable laser power. The time of acquisition was between 30 seconds and 1 minute per spot. The four MM10403188 spectra were imported into the MALDI BioTyper software (version 2.0, Bruker) and analyzed by standard pattern matching (with default parameter settings) against the main spectra of 3,769 bacteria including 129 spectra from 98 *Bacillus* species, notably *B. humi*, used as reference data, in the BioTyper database. The method of identification included the m/z from 3,000 to 15,000 Da. For every spectrum, 100 peaks at most were taken into account and compared with spectra in the database. A score enabled the presumptive identification and discrimination of the tested species from those in the database: a score > 2 with a validated species enabled the identification at the species level, a score > 1.7 but < 2 enabled the identification at the genus level; and a score < 1.7 did not enable any identification. For strain MM10403188^T^, the obtained score was 1.2, thus suggesting that our isolate was not a member of a known species. We incremented our database with the spectrum from strain MM10403188 (Figure 4). The spectrum was made available online in our free-access URMS database [24].

## Genome sequencing information

### Genome project history

The organism was selected for sequencing on the basis of its phylogenetic position and 16S rRNA similarity to other members of the genus *Bacillus*, and is part of a “culturomics” study of the human digestive flora aiming at isolating all bacterial species within human feces. It was the 60^th^ genome of a *Bacillus* species and the first genome of *Bacillus timonensis* sp. nov. A summary of the project information is shown in Table 2. The Genbank accession number is CAET00000000 and consists of 146 contigs.

**Table 2:** Project information

**MIGS ID**	**Property**	**Term**
MIGS-31	Finishing quality	High-quality draft
MIGS-28	Libraries used	454 GS shotgun and paired-end 3- kb libraries
MIGS-29	Sequencing platform	454 GS FLX Titanium
MIGS-31.2	Sequencing coverage	19×
MIGS-30	Assemblers	Newbler version 2.5.3
MIGS-32	Gene calling method	PRODIGAL
	INSDC ID	112529
	Genbank Date of Release	February 28^th^, 2012
	Gold ID	Gi13534
	NCBI project ID	CAET00000000
MIGS-13	Project relevance	Study of the human gut microbiome

### Growth conditions and DNA isolation

*B. timonensis* sp. nov. strain MM10403188^T^, CSUR P162, DSM 25372, was grown aerobically on 5% sheep blood-enriched BHI agar at 37°. Four petri dishes were spread and growth from the plates was resuspended in 3x500µl of TE buffer and stored at 80°C. Then, 500µl of this suspension were thawed, centrifuged 3 minutes at 10,000 rpm and resuspended in 3x100µL of G2 buffer (EZ1 DNA Tissue kit, Qiagen). A first mechanical lysis was performed by glass powder on the Fastprep-24 device (Sample Preparation system, MP Biomedicals, USA) using 2x20 seconds cycles. DNA was then treated with 2.5µg/µL lysozyme (30 minutes at 37°C) and extracted using the BioRobot EZ1 Advanced XL (Qiagen). The DNA was then concentrated and purified using the Qiamp kit (Qiagen). The yield and the concentration was measured by the Quant-it Picogreen kit (Invitrogen) on the Genios Tecan fluorometer at 50ng/µl.

### Genome sequencing and assembly

DNA (5 µg) was mechanically fragmented on a Hydroshear device (Digilab, Holliston, MA,USA) with an enrichment size at 3-4kb. The DNA fragmentation was visualized through the Agilent 2100 BioAnalyzer on a DNA labchip 7500 with an optimal size of 3.345kb. The library was constructed according to the 454 GS FLX Titanium paired-end protocol. Circularization and nebulization were performed and generated a pattern with an optimum at 492 bp. After PCR amplification through 15 cycles followed by double size selection, the single stranded paired end library was then quantified on the Quant-it Ribogreen kit (Invitrogen) on the Genios Tecan fluorometer at 339 pg/µL. The library concentration equivalence was calculated as 12,6E+08 molecules/µL. The library was stored at -20°C until further use.

The shotgun library was clonally amplified with 3cpb and the paired-end library was amplified with lower cpb (1 cpb) in 4 emPCR reactions with the GS Titanium SV emPCR Kit (Lib-L) v2 (Roche). The yields of the emPCR was 5.97% for the shotgun and 15.92% for the paired end as expected by the range of 5 to 20% from the Roche procedure.

Approximately 790,000 beads for a 1/4 region and 340,000 beads for a 1/8 region were loaded on the GS Titanium PicoTiterPlate PTP Kit 70×75 and sequenced with the GS FLX Titanium Sequencing Kit XLR70 (Roche). The run was performed overnight and then analyzed on the cluster through the gsRunBrowser and Newbler assembler (Roche). For the shotgun sequencing, 112,962 passed filter wells were obtained and generated 34.48Mb with a length average of 322 bp. For the shotgun sequencing, 213,882 passed filter wells were obtained and generated 50.6 Mb with a length average of 236 bp. The passed filter sequences were assembled Using Newbler with 90% identity and 40bp as overlap. The final assembly identified 11 scaffolds and 89 contigs (>1500bp) generating a genome size of 4.6 Mb.

### Genome annotation

Open Reading Frames (ORFs) were predicted using Prodigal [25] with default parameters but the predicted ORFs were excluded if they were spanning a sequencing gap region. The predicted bacterial protein sequences were searched against the GenBank database [26] and the Clusters of Orthologous Groups (COG) databases using BLASTP. The tRNAScanSE tool [27] was used to find tRNA genes, whereas ribosomal RNAs were found by using RNAmmer [28] and BLASTn against the GenBank database. ORFans were identified if their BLASTP *E*-value was lower than 1e-03 for alignment length greater than 80 amino acids. If alignment lengths were smaller than 80 amino acids, we used an *E*-value of 1e-05. Such parameter thresholds have already been used in previous works to define ORFans.

To estimate the mean level of nucleotide sequence similarity at the genome level between *Bacillus* species, we compared the ORFs only using BLASTN and the following parameters: a query coverage of ≥ 70% and a minimum nucleotide length of 100 bp.

## Genome properties

**Figure 5:**
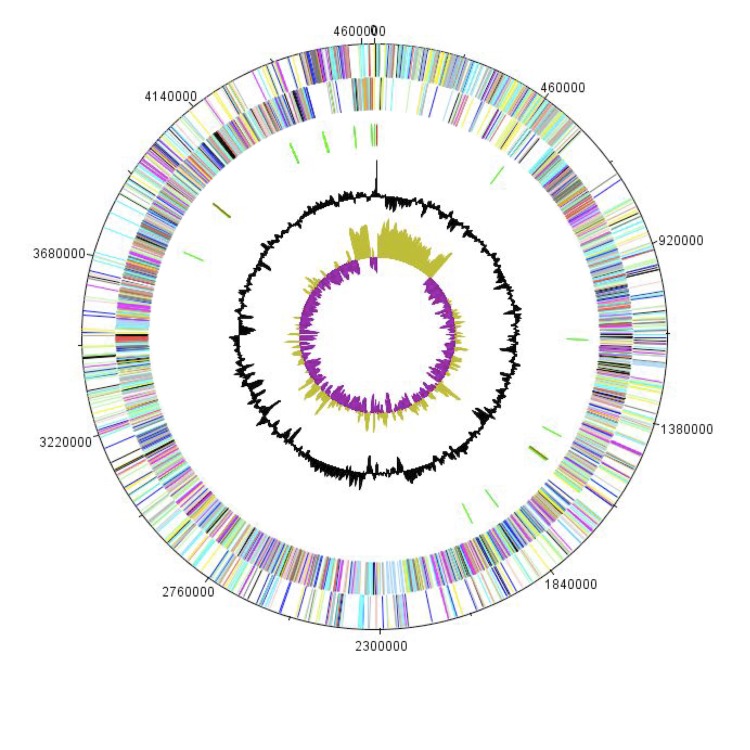
Graphical circular map of the chromosome. From outside to the center: Genes on the forward strand (colored by COG categories), genes on the reverse strand (colored by COG categories), RNA genes (tRNAs green, rRNAs red), GC content, and GC skew.

The genome is 4,632,049 bp long (1 chromosome, but no plasmid) with a 37.30% GC content (Figure 5 and Table 3). Of the 4,684 predicted genes, 4,610 were protein-coding genes and 74 were RNAs. A total of 3,399 genes (75.56%) were assigned a putative function. Three hundred forty genes were identified as ORFans (7.4%). The remaining genes were annotated as hypothetical proteins. The properties and the statistics of the genome are summarized in Tables 3. The distribution of genes into COGs functional categories is presented in Table 4.

**Table 3:** Nucleotide content and gene count levels of the genome

Attribute	Value	% of total^a^
Genome size (bp)	4,632,049	
DNA Coding region (bp)	3,959,694	85.48
DNA G+C content (bp)	1,727,754	37.3
Total genes	4,684	100
RNA genes	74	1.58
Protein-coding genes	4,610	98.42
Genes with function prediction	3,643	77.75
Genes assigned to COGs	3,399	75.56
Genes with peptide signals	189	4.03
Genes with transmembrane helices	1,261	26.92

^a^ The total is based on either the size of the genome in base pairs or the total number of protein coding genes in the annotated genome

**Table 4:** Number of genes associated with the 25 general COG functional categories

**Code**	**Value**	**% age**^a^	**Description**
J	181	3.93	Translation, ribosomal structure and biogenesis
A	0	0	RNA processing and modification
K	310	6.72	Transcription
L	169	3.67	Replication, recombination and repair
B	1	0.02	Chromatin structure and dynamics
D	39	0.85	Cell cycle control, mitosis and meiosis
Y	0	0	Nuclear structure
V	71	1.54	Defense mechanisms
T	193	4.19	Signal transduction mechanisms
M	197	4.27	Cell wall/membrane biogenesis
N	67	1.45	Cell motility
Z	0	0	Cytoskeleton
W	0	0	Extracellular structures
U	49	1.06	Intracellular trafficking and secretion
O	114	2.47	Posttranslational modification, protein turnover, chaperones
C	184	3.99	Energy production and conversion
G	349	7.57	Carbohydrate transport and metabolism
E	412	8.94	Amino acid transport and metabolism
F	97	2.10	Nucleotide transport and metabolism
H	121	2.62	Coenzyme transport and metabolism
I	150	3.25	Lipid transport and metabolism
P	245	5.31	Inorganic ion transport and metabolism
Q	100	2.17	Secondary metabolites biosynthesis, transport and catabolism
R	594	12.89	General function prediction only
S	361	7.83	Function unknown
-	606	13.15	Not in COGs

^a^ The total is based on the total number of protein coding genes in the annotated genome

## Comparison with the genomes from other *Bacillus* species

Genome sequences are currently available for more than 25 validly named *Bacillus* species. Here we compared the genome sequence of *B. timonensis* strain MM10403188^T^ with that of *B. licheniformis* strain ATCC 14580, the most closely related phylogenetic neighbor for which the genome sequence is available. The draft genome sequence of *B. timonensis* is larger than *B. licheniformis* (4.6 Mb and 4.2 Mb, respectively) but its G+C content is lower (37.30 and 46.19%, respectively). *B. timonensis* has more predicted genes than *B. licheniformis* (4,684 and 4,356, respectively), and more genes assigned to COGs (3,399 and 3,130, respectively). However, the distribution of genes into COG categories (Table 4) was highly similar in both genomes. In addition, *B. timonensis* shared a mean 86.10% (range 76.4-93%) sequence similarity with *B. licheniformis* at the genome level. 

Although the degree of 16S rRNA similarity was elevated (98.2%) between strain MM10403188 and *B. humi* strain DSM 16318, both strains exhibited several phenotypic and genomic differences, and we formally propose the creation of *Bacillus timonensis* sp. nov. that contains the strain MM10403188^T^. This strain has been found in Senegal.

### Description of *Bacillus timonensis* sp. nov.

*Bacillus timonensis* (tim.on.en′sis. L. gen. masc. n. *timonensis*, of Timone, the name of the hospital where strain MM10403188^T^ was cultivated.) Isolated from stool from an asymptomatic Senegalese patient. *B. timonensis* is an aerobic Gram-negative bacterium. Grows on axenic medium at 37°C in an aerobic atmosphere. Colonies were 3 mm in diameter on blood-enriched BHI agar. Cells grown on agar are sporulated and have a mean diameter of 0.66 µm. A positive reaction was obtained for L-arabinose, D-lactose, D-melibiose, D-trehalose, D-saccharose, and D-turanose fermentation. Positive reactions were obtained for oxidase, esterase, α-chimotrypsine, β-glucorinidase, and α- and β-glucosinidase activity. No catalase activity was exhibited. Positive for indole. By comparison with *B. humi*, *B. timonensis* differs in Gram staining, in culture atmosphere, as *B. humi* grows anaerobically, in catalase activity, in spore forming capacity, in indole production, and in carbohydrate metabolism, notably for arbutin, salicin, L-arabinose, melibiose, turanose, and trehalose. *B. timonensis* is susceptible to penicillin G, amoxicillin, vancomycin, gentamicin, erythromycin, doxycyclin, rifampicin, and ciprofloxacin but resistant to trimethoprim/sulfamethoxazole. Motile. The G+C content of the genome is 37.30%. The 16S rRNA and genome sequences are deposited in GenBank under accession numbers JF824810 and CAET00000000, respectively.

The type strain MM10403188^T^ (= CSUR P162 = DSM 253720) was isolated from the fecal flora of a healthy patient from Senegal.
