# Correction to: Non contiguous-finished genome sequence and description of *Bacillus timonensis* sp. nov

**DOI:** 10.1186/s40793-022-00448-8

**Published:** 2023-06-07

**Authors:** Sahare Kokcha, Ajay Kumar Mishra, Jean-Christophe Lagier, Matthieu Million, Quentin Leroy, Didier Raoult, Pierre-Edouard Fournier

**Affiliations:** Unité de Recherche sur les Maladies Infectieuses et Tropicales Emergentes, UMR CNRS 6236 ? IRD 198, Aix-Marseille Universit?, Marseille, France

**Correction: Standards in Genomic Sciences 20126:3**

10.4056/sigs.2776064.

Following publication of the original article [1], the authors flagged the following errors: The original version of this article unfortunately contained two mistakes: (1) the authors had omitted to mention 10403188T as type strain name of the new species *Bacillus timonensis* (MM10403188T had been written instead), and (2) in the last paragraph of the protologue, DSM 253720 had been written in place of DSM 25372.

The original article has since been updated to correct these errors. The authors thank you for reading and apologize for any inconvenience caused.
